# Seroprevalence of and risk factors for Q fever in dairy and slaughterhouse cattle of Jimma town, South Western Ethiopia

**DOI:** 10.1186/s12917-020-02598-8

**Published:** 2020-10-12

**Authors:** Feyissa Begna Deressa, David Onafruo Kal, Benti Deressa Gelalcha, Ricardo J. Soares Magalhães

**Affiliations:** 1 School of Veterinary Medicine, College of Agriculture and Veterinary Medicine, Jimma University, P.O.Box: 307, Jimma, Ethiopia; 2 Bahr El Ghazal University College of Veterinary Science, P.O. Box 10739, Wau, South Sudan; 3 UQ Spatial Epidemiology Laboratory, School of Veterinary Science, The University of Queensland, Gatton, Queensland 4343 Australia; 4 Children’s Health and Environment Program, Child Health Research Centre, The University of Queensland, South Brisbane, QLD 4101 Australia

**Keywords:** Cattle, Q fever, Seroprevalence, Ethiopia

## Abstract

**Background:**

Q fever is a zoonotic disease, caused by Gram negative bacterium *C. burnetii,* which imparts significant socio-economic burden due to production and reproductive loss (abortion, stillbirth, and infertility) in ruminants and debilitating clinical disease in human populations. While sheep and goats are considered the primary reservoirs of infection to humans, infection can also result from exposure to cattle. Recent studies indicate that in Ethiopia Q fever is a disease of growing public health interest. The top cattle producing region in Ethiopia is the Oromia region and Jimma is the zone that ranks first in the population of cattle within Oromia. While in Jimma zone livestock production plays an important role in people’s livelihoods and nutrition, to date, there is no available report on seroprevalence of Q fever in cattle. This is particularly important due to the low dairy farm biosecurity in Jimma town. This study aimed to evaluate the potential risk for public health from cattle production; a specific objective of this study included the estimation of the seroprevalence of *C. burnetii* infection and its potential risk factors in dairy cattle and cattle for slaughter in Jimma Town.

**Results:**

The seroprevalence of *C. burnetii* in cattle present at dairy farms was significantly lower compared to cattle presented at slaughterhouse [6.17% (95% CI: 3.41–10.13) and 11.79% (95% CI: 7.63–17.17), respectively; (*P* = 0.04)]. As the age of dairy cattle increase by 1 year, they were 1.51 more likely to be positive of *C. burnetii* [OR = 1.51 (95%CI: 1.30–1.75; (*P* = 0.000)]. Cattle managed in semi-intensive production systems were 8.08 more likely to be *C. burnetii* seropositive compared to intensively managed dairy cattle [OR = 8.08 (95%CI: 1.03–63.68); *P* = 0.047]. Dairy cattle with access to nuisance animals like dogs, cats and mice were 5.65 more likely to be *C. burnetii* seropositive compared to dairy cattle without access to these animals. On the other hand, dairy cattle that have no tick infestation are 93% less likely to be seropositive for *C. burnetii* [OR = 0.07 (95%CI: 0.01–0.74); *P* = 0.027]. Concerning farm-level data, farms of larger herd sizes were 1.03 more likely to be *C. burnetii* seropositive than small herd farms [OR = 1.03 (95%CI: 0.99–1.06)]. The result from slaughterhouse indicates that as the age of cattle increase by 1 year their chance of being *C. burnetii* seropositive increases by 2.27 [OR = 2.27 (95%CI: 1.93–2.68); *p* = 0.000].

**Conclusion:**

Considering its zoonotic and economic burden the seroprevalence of Q fever recorded in this study is of eminent public health concern with a farm-level and slaughterhouse seroprevalence of 6.17 and 11.79% respectively. Based on modifiable risk factors identified in this study, Q fever management plans better be focused on health education and awareness campaigns for abattoir workers and dairy farm workers. Dairy farm Q fever management plans should contemplate improved dairy herd biosecurity with regards to cattle tick infestation, keeping different livestock species segregated and avoiding mixing of herd with others with unknown health status.

## Background

Q fever is caused by highly infectious, ubiquitous and pleomorphic intracellular Gram-negative bacterium name *C. burnetii.* The organism can persist in a spore-like form for more than 40 months [11, 40]*.* The disease is classified as an emerging zoonotic infectious disease according to WHO, FAO, OIE and EFSA/ECDC [3, 15]. Sheep and goats are considered to be the major sources of human outbreaks due to *Coxiella,* but cattle can also be an important reservoir of the agent to humans [45].

Q fever has long been considered as an occupational zoonosis of major socio-economic importance worldwide associated with exposure to livestock by farmers, veterinarians, slaughterers, and animal researchers [56]. Its outbreaks have been occasionally observed in many countries throughout the world [14, 16, 27, 46, 55]. Despite the fact that the disease is widely distributed, the disease is regarded as neglected, under diagnosed and underreported because of its diverse symptoms, self-limiting course and lack of diagnostic tools [3, 7].

In the African Context Q fever was first reported in 1947, but since then the quantity and quality of epidemiological research on this pathogen has been limited [30]. Ethiopia was ranked highest in Africa in the health burden of zoonotic diseases [19]. The first evidence of *C. burnetii* was reported in ticks collected from cattle in Ethiopia [41]. As well as seroprevalence of *C. burnetii* was found to be 6.5% by complement fixation test in workers at Addis Ababa abattoir in goat and sheep slaughterhouses and its peri-urban zone as found by [1]. To date, the only study in Ethiopian concerning cattle was conducted in southeast of the country using enzyme-linked immunosorbent assay (ELISA) by [20]. This reported a high seroprevalence of *C. burnetii*, (31.6% in cattle, 90.0% in camels and 54.2% in goats). A 6.4% prevalence of *C. burnetii* in Ethiopia was also report from different Ixodid ticks species by quantitative real time polymerase chain reaction targeting two different genes followed by multi-spacer sequence typing (MST) by [28].

In recent years, reports of abortion and infertility in domestic ruminants from different corners of Ethiopia are becoming a common concern [2, 21, 33]. The Jimma zone in the Oromia Region of Ethiopia is one of such areas in from 2013 to 2015 it faced the worst outbreak of abortion, whereby more than 11,487 cases were recorded in domestic ruminants (cattle, goats and sheep) (Jimma zone livestock health and production agency, 2015). The Oromia Region of Ethiopia is the region with the highest population of cattle in the country and the Jimma zone of Oromia Region is the main cattle producing zone in Oromia and the second in Ethiopia with an estimated cattle population of 2,090,000 [17]. The unusually high losses of pregnancies and the resultant infertility in cattle within the Oromia region represent a tremendous economic loss to the nation and it is also a significant blow to the livelihoods of livestock producers in Ethiopia. The initial suspicions of *Brucella* involvement as a cause of the abortion cases in Oromia was ruled out by [12]. *Coxiella* was suspected to be one of the potential causes of such abortion episodes, as it can affect all three ruminant species. Nevertheless, to date there was no empirical evaluation of the level of seropositivity of cattle to *C. burnetii* in this important cattle producing zone of Ethiopia.

In this study, we aimed to identify the public health risk of *C. burnetii* to dairy farmers and communities in Jimma town in the Oromia region of Ethiopia with the objective of estimating the seroprevalence of *C. burnetii* and associated risk factors in cattle at Jimma dairy farms and its main slaughterhouse.

## Results

A total of 227 and 195 samples were collected from Jimma’s dairy farms and slaughterhouse respectively. The overall seroprevalence was 8.77% (95%CI: 6.07–11.47); *C. burnetii* seropositivity was significantly lower in dairy farms (6.17%; 95% CI: 3.41–10.13) compared to slaughterhouse (11.79%; 95%CI: 7.27–16.32%) (*p*-value≤0.042).

### Dairy farm-level C. burnetii seropositivity and its risk factors

Out of 227 animals included in the dairy farm analysis, the majority [*n* = 129 (56.83%)] originated from intensive management system and the vast majority were female [*n* = 223 (98.24%)]. Concerning their breed, the majority [*n* = 220 (96.92%)] were crossbred (Table 1) and in terms of age the minimum age sampled was 6 months and the maximum was 10 years. There was also higher seropositivity to *C. burnetii* in male cattle compared to female and higher seroprevalence in adult cattle compared to young. Prevalence of *C. burnetii* is found to be higher in the semi-intensive management system (8.16%; 95%CI: 3.59, 15.45) than in the intensive management system (4.65%; 95%CI: 1.73, 9.85) of dairy farms (Table 1).

**Table 1:** Univariable logistic regression analysis (adjusted for herd effect) to select forward factor for final model contributing to *C. burnetii* distribution in dairy cattle and slaughter cattle of Jimma, Ethiopia (*n* = 227; 195 respectively)

Variable	Category	No tested	Prevalence (%)	95%CI		OR(95% CI)	*P*- value
				Lower	Upper		
Age^a^	In years	227	14 (6.17)	3.41	10.13	1.33(1.04–1.69)	0.021
Sex	Male	4	3(75.0)	19.41	99.37	57.25(10.29, 318.50)	0.000
	Female	223	11(4.93)	2.49	8.65	1	
breed	local	7	1(14.29)	0.36	57.87	2.71(0.21, 34.95)	0.444
	Crossholisten	220	13(5.91)	3.18	9.89	1	
BCS^b^	Ordinal scale	57	2(3.51)	0. 43	12.11	1	0.243
Multiage mix	No	115	8(6.96)	3.05	13.25	1.33(0.52,3.43)	0.548
	Yes(ref)	112	6(5.36)	1.99	11.30	1	
MultiSpecies mix	yes	203	14(6.90)	3.82	11.30	∞	0.000
	No	24	0(0.0)	0.00	14.25	1	
Tick infest	No (Ref)	123	7(5.69)	2.32	11.37	1	0.746
	Yes	104	7(6.73)	3.30	13.25	1.21(0.08, 18.30)	
Herd size^c^	Continuous scale	227	14 (6.17)	3.41	10.13	1.01(0.99, 1.03)	0.163
Contact other herd	No	206	12(5.83)	3.05	9.95	1	0.477
	yes	21	2(9.52)	1.17	30.38	1.71(0.38, 7.51)	
Management system	Intensive	129	6(4.65)	1.73	9.85	1	0.170
	Semi-intensive	98	8(8.16)	3.59	15.45	1.84(0.77,4.41	
Presence nuisance animals (dog,cat, mice…)	No	70	3(4.29)	0.89	12.02	1	0.314
	Yes	157	11(7.01)	3.55	12.19	1.71(0.60,4.82)	
Total	cattle	227	14 (6.17)	3.41	10.13		
**Female data (n = 223)**							
Animal aborted	No(ref)	191	9(4.71)	2.18	8.76	1	0.722
	Yes	32	2(6.25)	0.77	20.71	1.35(0.26, 6.98)	
Parity	Heifer (ref)	65	2(3.08)	0.37	10.68	1	
	Perimiparous	42	3(7.14)	1.50	19.48	2.43(0.26, 22.34)	0.435
	Multiparous	116	6(5.17)	2.39	10.83	1.72(0.39,7.62)	0.476
**Slaughterhouse data (*n*** **= 195)**							
Age	In years	195	23 (11.79)	7.63	17.17	6.93(3.51, 13.66)	0.000
BCS^b^	Ordinal scale	195	23 (11.790	7.63	17.17	0.48(0.24–0.99)	0.049
Tick infest	No (Ref)	22	2 (9.09)	1.12	29.16	1	0.678
	Yes	173	21 (12.14)	7.67	17.96	1.38 (0.30–6.36)	
Total	cattle	195	23 (11.79)	7.63	17.17		

Legend: *Ref*. Reference, *OR* Odds Ratio, *CI* Confidence Interval, ^a^Age was measured in years, ^b^BSC Body condition Score on 9 ordinal scale, ^c^Herd size was the number of cattle in the farm

The final animal-level multivariable logistic regression mixed effect model showed that *C. burnetii* seropositivity is significantly positively associated with age (OR: 1.51(95%CI: 1.30, 1.75): *p*-value≤0.000) (Table 2). Our results also show that cattle managed in semi-intensive system were 8.08 more likely to be *C. burnetii* seropositive compared to intensively managed dairy cattle [OR = 8.08 (95%CI: 1.03, 63.68); *P* = 0.047]. Dairy cattle that have access to nuisance animals like dogs, cats, mice and other were 5.65 more likely to be *C. burnetii* seropositive compared to dairy cattle with no access to nuisance animals (Table 2). On the other hand, dairy cattle that have no tick infestation are 93% less likely to be seropositive for *C. burnetii* [OR = 0.07 (95%CI: 0.01, 0.74); *P* = 0.027] (Table 2).

**Table 2:** Results of final best fitting multivariable mixed effect generalized linear model for the probability of *C. burnetii* seropositivity in dairy cattle (n = 227) in Jimma, Ethiopia

Variables	Category	No tested	Prevalence (%)	95%CI		OR(95% CI)^a^	*P*- value
				Lower	Upper		
Age^b^	In years	227	14 (6.17)	3.41	10.13	1.51 (1.30,1.75)	0.000
Tick infest	No (Ref)	123	7(5.69)	2.32	11.37	1	0.027
	Yes	104	7(6.73)	3.30	13.25	0.07(0.01, 0.74)	
Management system	Intensive	129	6(4.65)	1.73	9.85	1	0.047
	Semi-intensive	98	8(8.16)	3.59	15.45	8.08(1.03,63.68)	
Presence nuisance animals (dog, cat, mice…)	No	70	3(4.29)	0.89	12.02	1	0.120
	Yes	157	11(7.01)	3.55	12.19	5.65(0.64,50.23)	
Parity	Heifer (ref)	65	2(3.08)	0.37	10.68	1	
	Perimiparous	42	3(7.14)	1.50	19.48	0.56(0.07, 4.39)	0.580
	Multiparous	116	6(5.17)	2.39	10.83	0.45(0.11, 1.88)	0.272

Legend: *Ref*. Reference, *OR* Odds Ratio, *CI* Confidence Interval, ^a^Adjusted for random effect of farm, ^b^Age was measured in years

Out of twenty-five dairy farms sampled, seven of them had at least one infected animal resulting in a herd-level *C. burnetii* seropositivity of 28% (95%CI: 12.07–49.39). Dairy farms which had at least one contact with other herds were 4.63 time more likely *C. burnetii* seropositive than herd which had no contact [OR = 4.63 (95%CI: 0.79, 26.94)] but that difference was marginally significant (Table 3).

**Table 3:** Multivariable Binomial Generalized linear models of factors at farm level (*n* = 25 farms) for *C. Burnetii* sero-distribution in dairy cattle of Jimma, Ethiopia

Variable	Category	No tested	Prevalence (%)	95%CI		OR(95% CI)	*P*- value
				Lower	Upper		
Herd size	small	6	0(0.0)	0.00	45.93	ref	0.120
	Large	19	7(36.84)	16.29	61.64	1.03 (0.99, 1.06)	
Contact other herd	No	21	5(23.81)	8.22	47.17	ref	0.088
	yes	4	2(50.00)	6.76	93.24	4.63 (0.79, 26.94)	
Management system	Intensive	20	5(25.00)	8.66	49.10	ref	0.348
	Semi-intensive	5	2(40.00)	5.27	85.34	2.94 (0.31,25)	
Total	cattle	25	7 (28)	12.07	49.39		

Legend: *OR* Odds Ratio, *CI* Confidence Interval, *ref*. Reference

### Slaughterhouse animals’ C. burnetii seroprevalence and their risk factors

The overall seroprevalence of *C. burnetii* antibodies from cattle sampled at slaughterhouse was found to be 11.79% (95%CI: 7.63, 17.17). Out of 195 animals included in the slaughterhouse analysis, all were from extensive management system, males and local breeds. All *C. burnetii* seropositive cattle were adults. Prevalence of *C. burnetii* antibody was found to be higher in tick infested cattle (12.14%) than the non-tick infested cattle (9.09%). Higher prevalence was recorded in medium body conditioned (16.22%) cattle compared to good body conditioned cattle (9.09%) (Table 1).

In the multivariable model of animals sampled at slaughterhouse age of cattle was the only factor found to be associated with *C. burnetii* seropositivity [OR = 2.27 (95%CI: 1.93, 2.68); *p* = 0.000], which means as age of cattle increase by 1 year, their chance of being *C. burnetii* seropositive increases by 2.27.

## Discussion

This research is the first to investigate the seroprevalence of and risk-factors for *C. burnetii* exposure in cattle in Jimma Town the most important city in the second highest cattle production zone of Ethiopia. The i-ELISA test used was claimed to have 100% sensitivity (Se) and specificity (SP) as described by the manufacturer using serum from confirmed infected animals but other authors cited the test sensitivity and specificity for serum as 100 and 95%, respectively, compared to PCR [18]. Overall our results demonstrate that *C. burnetii* infection is a significant public health problem in the area in that 8.77% (95%CI: 6.07–11.47) of tested animals were found with evidence of *C. burnetii* antibodies. Our results suggest that cattle in Jimma town have a high level of exposure to *C. burnetii* infection which could partly explain the observed reproductive disorders and abortions occurring in Jimma zone. Our findings are in agreement with 7.9% report in Algeria (sample size 311, cross sectional and tested with ELISA) [8], but higher than similar studies undertaken in Bura, Tana River County, Kenya which reported 5% (Sample size 96, cross sectional study design and ELISA test) [35], and 4% in Chad (sample size 195, cross sectional with i-ELISA) [49]. However, the overall seroprevalence reported in our study is lower compared to the previous studies in the Southeast Ethiopia (i.e. a seroprevalence of 31.6% using cELISA) by [20], and other countries in Africa ranging between 13 and 32% [25, 26, 36, 38, 39, 50]. The possible reasons for these variations might be the difference in sample sizes, sampling methods and diagnostic tests used, geographical locations and management systems being practiced. Our results indicate that *C. burnetii* seroprevalence in cattle in Jimma town is significantly higher in cattle sent to slaughterhouse compared to dairy cattle in dairy farms (11.79% vs 6.17%) suggesting that management systems may play an important role at modulating exposure risk [6]. This might partly be explained by the fact that all cattle sampled at the slaughterhouse were local breeds kept under extensive management systems from a variety of different districts of Jimma zone. This finding is in line with the study conducted in Nigeria which reported a prevalence of 11% in cattle at slaughterhouse and a prevalence of 17.1 and 1.3% in local breed and cross breed respectively [54]. Extensive management systems allow for an increase in exposure opportunities to *C. burnetii* through aerosol transmission between animals at grazing and watering areas. The extensive management system also exposes cattle to wildlife which could play a relevant role for disease species cross-transmission [47].

Similarly, for dairy cattle our results indicate a significantly increased probability of seropositivity in crossbred dairy cattle kept under semi-intensive dairy production compared to intensive management system of dairy production. Cross breed dairy cattle are expensive and mostly kept under either intensive or semi-intensive management systems so that disease and tick are better controlled. Further, *C. burnetii* can survive in dry dusty environmental conditions for months and cattle managed in a semi-intensive system can be at greater risk of exposure to contaminated aerosols from known transmission vehicles from infected animals such as urine, feces or birthing products in the field compared to cattle managed in intensive systems [9]. This is in agreement with other studies showing that dairy cows which were partially grazing in the field had higher seropositivity to *C. burnetii* antibodies [6]. In addition, our study demonstrated that dairy cattle with access to nuisance animals (such as dogs, cats, mice) were more likely to be seropositive to *C. burnetii* antibodies compared to dairy cattle with no access to nuisance animals. This finding is supported by evidence suggesting the ability for a range of companion animals and pests to be infected with *C. burnetii* [4, 22, 32, 42, 43].

In our study we found a significant increase in the probability of *C. burnetii* exposure with increasing age in both dairy cattle and cattle sampled at slaughterhouse. This finding is in agreement with previous studies in Ethiopia and Cameroon [20, 30] and a more recent study by [24] describing the age distribution of *C. burnetii* antibodies in camels, cattle, goats and sheep. One possible explanation is that the older the animal the greater is the potential exposure to the pathogen infections and keep circulating antibody [5, 31]. On the other hand, the result indicates that dairy cattle with evidence of tick infestation had significantly higher increase in the probability of *C. burnetii* seropositivity (*p*-value≤0.027). This result is also in line with evidence from around the world pointing for the isolation of *C. burnetii* from ticks [23, 26, 28] indicating a potential role of tick infection in the dissemination of Q fever in the herd.

Our results indicate that dairy farm-level seroprevalence was marginally higher in farms with contact with other herds. Previous research reported that partial housing of the herds, contact with other herds and extensive management systems increased the likelihood of seropositivityto *C. burnetii* [6, 24, 48, 53, 57]. These are all modifiable farm-level bio-exclusion factors which can be acted upon by farmers to reduce the changes of *C. burnetii* transmission into the herd.

The findings of this study carry significant public health implications for the need to control Q fever in the community. Our results indicate that there is a significant risk of Q fever particularly in slaughterhouse workers, dairy farmers and other animal workers and the consumers of dairy products in Jimma town. Our results suggest that the burden of Q fever in these occupational groups identified is likely to be high and a collective effort is needed to investigate its impact on human health as well as to improve health promotion and education to these target community groups. Q fever awareness campaigns and on-farm Q fever biosecurity management plans need to be implemented in Jimma slaughterhouse workers and dairy cattle farmers with the aim of reducing their risk of exposure to *C. burnetii*. Furthermore, the level of seroprevalence demonstrated in dairy farms necessitates more attention because these animals are the milk source for children. The veterinarian and public health sector need to work together in a One health approach to investigate the shared burden of Q fever in the province of Oromia.

The findings of this study should be interpreted in light of its limitations. First, the cross-sectional nature of our investigation coupled with the use of serological tests for ascertainment of *C. burnetii* exposure means that we were unable to conclude on the true infection status of animals/herds. Second, from the slaughterhouse survey we were unable to include female cattle which are usually managed under extensive management systems and thereby provide a more complete epidemiological picture of the level of *C. burnetii* infection in rural population.

## Conclusion

The present study indicates that *C. burnetii* exposure is significantly high in cattle in an area in Ethiopia with one of the highest cattle populations in the country. Our findings demonstrate important modifiable farm-level risk factors which can be used to design farm-level Q fever biosecurity management plans and Q fever health promotion campaigns to reduce the public health of risk of *C. burnetii* exposure. Further studies should be designed to investigate the level of *C. burnetii* exposure in dairy farmers, slaughterhouse workers and consumers of dairy production in the region.

## Methods

### Study area and period

**Fig. 1:**
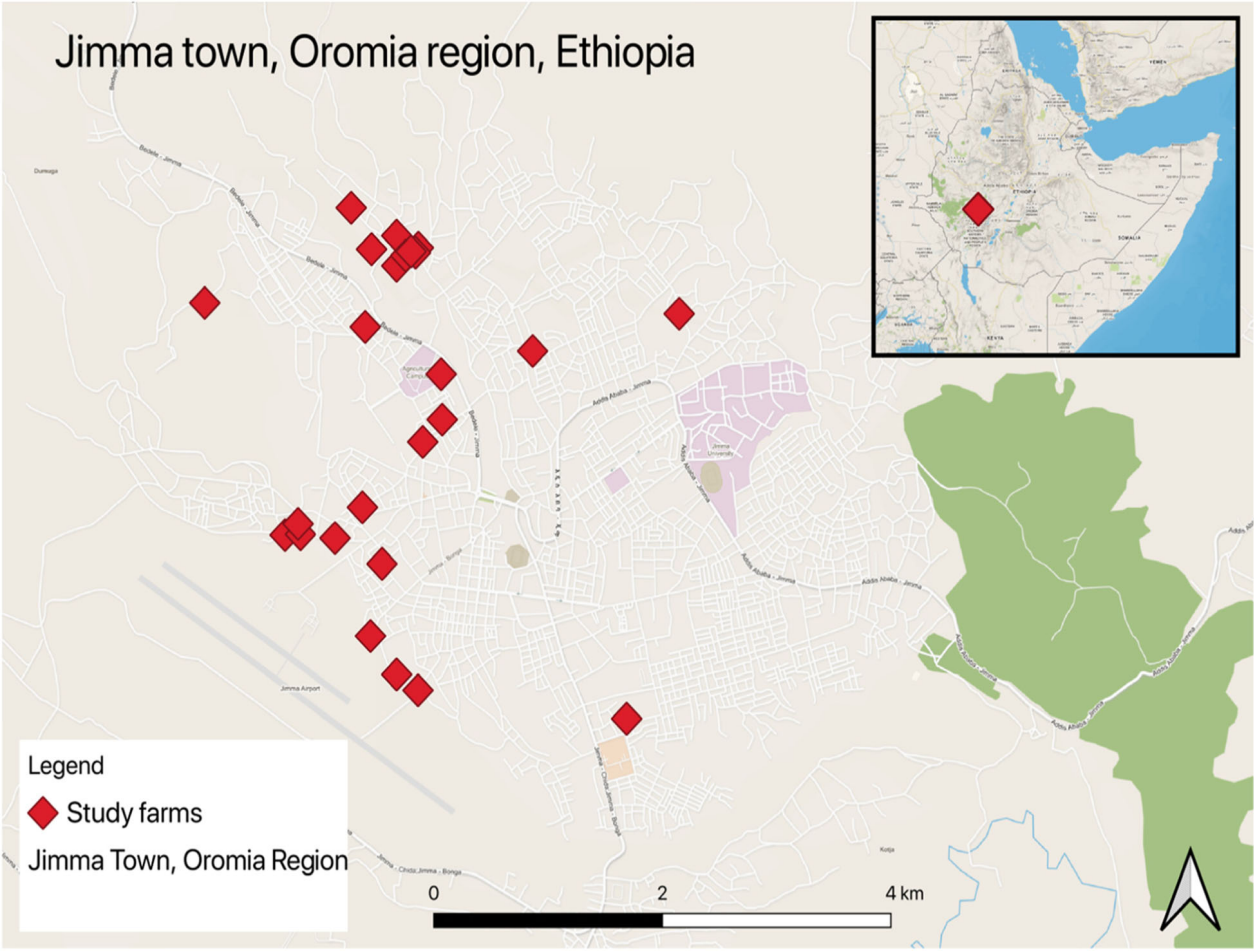
Study area and location of study farms (Map created by ArcGIS® software Esri version 10.8 (https://desktop.arcgis.com/en/arcmap/))

This study was conducted in Jimma Town from October 2016 to October 2017. The town is located in the Jimma zone of Oromia Regional State, South Western Ethiopia (Fig. 1). Jimma town is situated at a distance of 356Km, South West of Addis Ababa, the capital city of Ethiopia, between 7°41“N latitude and 36°50”E longitudes and has an altitude of 1704 m above sea level. The climate of the area is a tropical humid climate characterized by heavy rainfall which ranges from 1200 to 2000 mm per annum. With the mean annual minimum and maximum temperature ranging from 6 °C and 31 °C respectively, the overall average temperature is approximately 18.5 °C. Jimma zone is one of the largest in livestock populations in Ethiopia with cattle population estimated 2,212,962 heads [10]. Dairy cattle are more under production in Jimma town and the surroundings small towns but more than 95% of the cattle populations are under extensive management which are used for mixed dairy and meat production as well as cash income generation for the rural communities.

### Target and study population

The target population was apparently healthy crossbred dairy cattle kept under intensive and semi-intensive management systems and local breed cattle which are kept under extensive management system. These involved smallholder dairy farms and Jimma Dairy Development Enterprise (JDDE) and the local breed of male cattle presented to slaughterhouse aged between 3 and less than 10 years.

### Sample size determination

The sample size to arrive at the study population was determined using the formula described ((Z^2^ x P(1- P))/e^2^ where Z = 1.96 from normal distribution table, P = expected prevalence, e = desired precision level) by [13]. The conservative estimate of 50% prevalence, 95% level of confidence and 5% absolute precision was used. Accordingly, the estimated sample size of 384 animals was obtained. The calculated sample size was oversampled by 10% to account for possible problems with non-response or missing data [37]. This allowance was added summing up to the total of 422 samples. These samples were approximately halved to be distributed to dairy farms and slaughterhouse for blood sample collection. The proportion of required number of samples from each dairy farm was obtained by multiplying 28.3% expected prevalence of *C. burnetii* in cattle reported from Kenya [26] to the total number of cattle in each dairy farm; on average a total of 9 animals were sampled from each dairy farm.

### Study design and sampling strategy

Two cross sectional studies were designed to achieve the objectives of this study. First, a slaughterhouse survey was designed in the following way: in each day of visit to the slaughterhouse for a period of 2 weeks, a representative percentage of 25% of animals were picked by simple random sampling technique from the lairage during ante mortem inspection. The sampling frame was constructed by listing the total number of animals in the lairage of each visiting day. The total number of slaughtered animals in Jimma slaughterhouse ranged from 55 to 85 per day. On average, 14 samples were sampled per day to attain the total samples required (ie. 195 samples) and after sampling, animal level data like age, sex, tick infestation, breed, body condition score, production system were recorded.

Second, a farm-level survey was designed to measure Q fever exposure in the following way: a list of all 61 dairy farms and their contact details and location (ie. *Kebele*) was obtained from Jimma town livestock and fisheries resources development office. Thus a total of 25 dairy farms were selected by simple random sampling technique out of the 61 farms on the list to satisfy the total sample required from dairy farms. Each farm was visited once for about 1 month sampling period. All targeted farms are business oriented dairy farms with crossbred and/or pure exotic breeds of dairy cattle (Holstein- Friesian). Based on [34], herd size was categorized as small if the total number of animals in the herd was 3–10 animals, and large if the animal number in the herd were 11 or above. A farm owner questionnaire was used to collect risk factor data for Q fever infection, including individual-level data and farm-level data. For individual-level data animals’ age in years was recorded by means of dentition (as described by [29]) and also asking farm owners. Additional individual-level independent variables included sex, body condition score (BCS; categorized as poor, good and very good as described by [44]), breed, tick infestation status of animals and animal parity, and abortion status. For farm-level data these included multi species mix, multi age mix, history of contact with other herds, herd size (in a continuous numeric scale), production system (classified as intensive, semi-intensive and extensive), presence of nuisance animals in the farm (eg. presence of dogs, cats, rodents and others), were included in the questionnaire/check list (Additional file 1: Appendix 1**)**.

### Specimen collection procedure

About 10 ml of blood sample was collected from the jugular vein of each selected cattle using plain vacutainer tubes and multipurpose disposable blood collection needle 21Gx1 1/2″ plus needle holder (Zhejiang Kanshi) Medical Devices Co. Ltd. (HENSO). Before and after sample collection, 70% ethanol alcohol was applied as disinfectant. Each specimen was labeled with unique identification number. The tubes were transported to Jimma University College of Agriculture and Veterinary Medicine laboratory in an icebox and the tubes were put in an oblique position of 45°, for overnight at room temperature, to allow clotting of blood, the next morning sera was gently pipetted into cryovials and stored in deep freezer at − 20 °C, until diagnosis was made in the laboratory of National Veterinary Institute (NVI) at Debre-Zeit, Ethiopia.

### Laboratory analysis and interpretation

All serum samples were tested using Indirect Enzyme-Linked Immunosorbent Assay (i-ELISA) from ID Screen®Q fever Indirect Multi- Species kits (ID.vet, 310; rue Louis Pasteur–Grabels–France) for the detection of antibodies against *C. burnetii*. All reagents were prepared and results were interpreted according to the manufacturer’s instructions. Briefly, the optical densities (OD) were read at 450 nm in a micro-plate photometer (Multi Skan Ex, Thermo Electron Corporation, Finland). Negative control (NC), and positive control (PC) were run as duplicates in the micro–plate wells A, B and C, D respectively whereas sera were run as a single spot in the remaining micro plate wells. Interpretation of the result for each sample was obtained as the percentage of the ratio between the sample Optical Density (OD) and positive control OD, according to the $\frac{S}{P}\%$ formula as given below.

$$ \frac{S}{P}\%\frac{OD\; sample- OD\; negative\kern0.17em control}{{}^{-} OD\; positive\kern0.17em control- OD\; negative\kern0.17em control}x100. $$

The negative and positive samples were determined based on the laboratory test thresholds–values for its status (Table 4). The coloration quantity depends on the presence of antibodies in the specimen; positive sample will remain colored after addition of stop solution, while the light yellow negative sample will be colorless or white.

**Table 4:** Serum and plasma samples thresholds–values and status for the interpretation of ELISA test

Result	Status
S/P % ≤ 40%	Negative
40% < S/P % ≤ 50%	Doubtful
50% < S/P % ≤ 80%	Positive
S/P % > 80%	Strong positive

### Data management and statistical analysis

All data collected during the sero-surveys were entered into MS Office Excel 2010. Data were analyzed separately for cattle sampled in dairy farms and cattle sampled at the slaughterhouse. The overall prevalence was calculated as a total number of positive samples for *C. burnetii* divided by the total number of samples tested multiplied by 100. For each prevalence, binomial ‘exact’ 95% confidence interval (CI) was calculated using Epitools [51]. To statistically test the difference between the overall prevalence in dairy farms and slaughterhouse, a test for two sample proportions was calculated using the proportion test calculator in the statistical software STATA version 13 [52]

Univariable mixed effect logistic regression analysis was used to select individual explanatory variable that may predict individual *C. burnetii* seropositivity. Variables with a *p*-value < 0.25 at the univariable screening were taken forward to a multivariable mixed effect generalized linear model (farm as random effect) with Bernoulli family with a logit link. A separate multivariable binomial generalized linear model was used to model herd level prevalence data. Slaughterhouse data was analyzed using logit generalized linear model. Furthermore, multicollinearity was also assessed for any correlation between the explanatory variables with Spearman’s rank correlation and between management system and contact with other herds shows there is a correlation (Spearman’s rho = − 0.6001; *P*-value≤0.0015). Interaction terms between explanatory variables were entered into the model to investigate the presence of effect modification. Statistical significance in the multivariable model was set at a *P*–value ≤0.05. All statistical analyses were performed in Stata statistical software version 13 [52].

## Supplementary information

**Additional file 1.** Questionnaire/check list used during animal and risk factors data collection.

## Data Availability

The data supporting the findings of article are not available publicly due to ethical reason and are available from the corresponding author upon reasonable request.

## Abbreviations

21Gx1: 21 Needle Gauge
BCS: Body condition score
*C. burnetii*: *Coxiella burnetii*
CDC: Centers for Disease Control and Prevention
cELISA: Competitive Enzyme-linked Immunosorbent Assay
CI: Confidence interval
Co. Ltd.: Company Limited
CSA: Central Statistical Agency
E: East
ECDC: European Centre for Disease Prevention and Control
EFSA: European Food Safety Authority
FAO: Food and Agricultural Organization
ID.vet: Diagnostic kits producing company for farm animals; Louis Pasteur, France
I-ELISA: Indirect Enzyme-linked Immunosorbent Assay
JDDE: Jimma Dairy Development Enterprise
Km: Kilometer
MS: Microsoft
N: North
NC: Negative control
NVI: National Veterinary Institute
°C: Degree Celsius
OD: Optical densities
OIE: World organization for animal health
OR: Odds ratio
P: Predictive value (*p*-value)
PC: Positive control
PCR: Polymerase chain reaction
Q fever: Query fever
QLD: Queensland
Se: Sensitivity
SP: Specificity
UQ: The University of Queensland
WHO: World Health Organization
