# Supplementary material for: Seroprevalence of and risk factors for Q fever in dairy and slaughterhouse cattle of Jimma town, South Western Ethiopia
Source: BMC Vet Res. 2020 Oct 12;16:385. doi: 10.1186/s12917-020-02598-8 (PMC7552523; doi:10.1186/s12917-020-02598-8)
Supplement: Supplementary file 1 — Additional file 1. Questionnaire/check list used during animal and risk factors data collection. [file 12917_2020_2598_MOESM1_ESM.docx]

**Appendix 1: Questionnaire/check list used when the animal and risk factor data were collected**

1. Animal identification (animal ID)
2. Herd identification (herd ID)
3. Area of residence: Urban Peri - urban Rural
4. Age (in year)
5. Sex
6. Breed
7. Species
8. Body condition scores (5 scale)
9. Herd multi-age mix A. Yes B. No
10. Multi-species mix A. Yes B. No
11. Tick infestation A. Yes B. No
12. Herd size (number of animals in the farm)__________________________
13. Contact with other herds A. Yes B. No
14. Management system A. intensive C. semi intensive extensive
15. Health status (during sampling) A. clinical ill B. normal
16. Use of artificial insemination A. Yes B. No
17. Do you supply straw to your animal from other area? A. Yes B. No
18. Are their nuisance animals in your herd (e.g. wild birds, dogs, cats, rodents..)? A. Yes B. No
19. Have you ever encountered abortion in your herd? A. Yes B. No
20. If yes, how do you handle the aborted feotus/materials? Burn Buried Leave in open field
21. If sampled animal is female, did she abort? Yes No
22. How many times did the cow give birth/parity? ___________________________
23. Do you know anything about Q-fever in cattle? A. Yes B. No
24. How do you treat Q – Fever in animal: Traditional Medicine None
